# The Effects of Salvianolate Combined With Western Medicine on Diabetic Nephropathy: A Systematic Review and Meta-Analysis

**DOI:** 10.3389/fphar.2020.00851

**Published:** 2020-06-12

**Authors:** Yuehong Shen, Shulin Wang, Yuanyuan Liu, Ling Ge, Lili Xia, Xiaoxiao Zhang, Yuying Miao, Jianping Shen, Qian Zhou

**Affiliations:** ^1^ Nanjing University of Chinese Medicine, Nanjing, China; ^2^ Department of Orthopaedics, Zhenjiang City Hospital of Traditional Chinese Medicine, Zhenjiang, China; ^3^ Affiliated Hospital of Integrated Traditional Chinese and Western Medicine, Nanjing University of Chinese Medicine, Nanjing, China

**Keywords:** salvianolate, western medicine, diabetic nephropathy, traditional Chinese medicine, meta-analysis

## Abstract

**Background:**

Salvianolate, a compound mainly composed of salvia magnesium acetate, is extracted from the Chinese herb *Salvia miltiorrhiza*. Because of its biological activity, easy quality control and certain efficacy, salvianolate is widely used in treating ischemic cardiocerebral vascular disease, liver damage, renal injury, diabetes, and its complications. Particularly, it has potential protective effects on diabetic nephropathy (DN).

**Objective:**

This meta-analysis aimed to evaluate the efficacy and safety of salvianolate when combined with western medicine in patients affected with DN.

**Methods:**

We searched Pubmed, Web of Science, the Cochrane Library, China National Knowledge Infrastructure (CNKI), Wanfang Data knowledge service platform (Wanfang Data), Chinese Scientific Journal Database (VIP), and China Biology Medicine Disc (SinoMed) for randomized controlled trials (RCTs) of salvianolate in combination with western medicine on DN, including results from the foundation of each database until November 30, 2019. Two reviewers independently performed literature screening, data extraction, and quality evaluation. This meta-analysis was carried out using RevMan5.3 software.

**Results:**

From the 12 RCTs, 1,030 patients from China were involved. Compared with single-use western medicine, the combination of salvianolate and western medicine for the treatment of DN could reduce levels of serum creatinine (Scr) [MD=−16.53, 95% CI (−28.79, −4.27), *P*=0.008], blood urea nitrogen (BUN) [MD=−1.40, 95% CI (−2.17, −0.62), *P*=0.0004], urinary albumin excretion rate (UARE) [SMD=−1.84, 95% CI (−2.70, −0.98), *P* < 0.0001], 24-hour urinary protein (24h Upro) [MD=–0.37, 95% CI (–0.47, –0.26), *P* < 0.00001], albumin-to-creatinine ratio (ACR) [SMD=–1.43, 95% CI (–2.64, –0.23), *P*=0.02], hypersensitive C-reactive protein (hs-CRP) [MD=−5.69, 95% CI (−7.09, −4.29), *P* < 0.00001], interleukin-6 (IL-6) [MD=−12.53, 95% CI (−18.55, −6.52), *P* < 0.0001], malondialdehyde (MDA) [SMD=−2.05, 95% CI (−3.67, −0.43), *P*=0.01], as well as improve clinical efficacy [RR=1.21, 95% CI (1.12,1.31), *P* < 0.00001], and increase superoxide dismutase (SOD) levels [SMD=1.12, 95% CI (0.86,1.38), *P* < 0.00001]. No increase in the occurrence of serious adverse events were observed in the treatment group compared with the control group.

**Conclusion:**

This study indicated that salvianolate combined with western medicine contributes to protecting renal function, inhibiting inflammation, and exhibiting anti-oxidative properties, thereby improving clinical efficacy. Thus, salvianolate can be considered as a potential complementary therapy for DN patients. However, due to the low quality of methodology and small sample sizes, more rigorous and larger trials are essential to validate our results.

## Introduction

Diabetic nephropathy (DN) is the most common microvascular complication of diabetes with persistent microalbuminuria, renal tubules, and renal interstitial fibrosis as its main clinical features (Gross et al., 2005). Its incidence is 25%−40% in patients affected with type 1 diabetes, whereas it is 5%–40% in those with type 2 diabetes (American Diabetes Association, 2017). Patients with DN have increased risks of coronary heart disease, stroke, and peripheral artery disease. Approximately 50% of DN patients worldwide eventually reach end-stage renal disease (ESRD) without effective treatment (Ritz et al., 2011; Kanwar et al., 2011). Hyperglycemia is the main driving force of DN, causing kidney damage either directly or through hemodynamic changes (Schena and Gesualdo, 2005). Moreover, hypertension, dyslipidemia, elevated uric acid, obesity, and smoking accelerate DN progression (Suryavanshi and Kulkarni, 2017). Its pathogenesis may be linked to renal hyperfiltration and renal injury, glucose metabolic disorder, vasoactive substances, and heredity (Bhattacharjee et al., 2016). Chronic inflammatory response and oxidative stress also play important roles on DN and have become appropriate targets for therapeutic intervention (Tziomalos and Athyros, 2015; de Gaetano et al., 2018). According to Mogensen Stage, DN can be divided into five stages. Most patients were diagnosed in stage III or after stage III, because the symptoms were unconspicuous when in stage I and II (Badal and Danesh, 2015). Once DN enters to end stage (stage V), of which treatment would be more difficult than other kidney diseases (Damm et al., 2013). In consequence, interventions for delaying DN progression and improving the condition become particularly important. Currently, the key to treating DN is intensive glycemic control, and thus new antidiabetic drugs with specific renal protective effects are widely used in clinical practice (Moreno et al., 2018). Previous studies have discovered two major classes of antihypertensive drugs, i.e., angiotensin converting enzyme inhibitors (ACEIs) and angiotensin receptor blockers (ARBs), which are available to slow down renal disease progression by controlling albuminuria (Ajiboye and Segal, 2017). In spite of tremendous advances in the pharmaceutical drug industry, the condition of renal function in DN patients and the side effects of some drugs have restricted the clinical application of western medicine (Xiong et al., 2013). Thus, more novel and effective therapies should be seeked beyond active symptomatic treatment for patients affected with DN.

Traditional Chinese medicine (TCM), as therapeutic alternatives, has shown unique advantages on DN, owing to its characteristics of multicomponents and multitargets (Peng et al., 2020). *Salvia miltiorrhiza*, a famous Chinese herb, has been mentioned in the oldest Chinese book of material medica “Shennong Herbal Classic” with the reputation of “one *Salvia miltiorrhiza*, the same function as Siwu decoction”. Salvianolate is a compound preparation extracted from *salvia miltiorrhiza*, which mainly consists of salvia magnesium acetate (3 molecules of 3,4-dihydroxyphenyllactic acid and 1 molecule of caffeic acid, 80%) and homologous compounds (magnesium shikonate, sodium rosemary, salvia dipotassium acetate, potassium salvia, and dipotassium shikonate, 20%) (Wang and Xuan, 2005; Li et al., 2019). As a water-soluble ingredient, salvianolate possesses typical bioactivities such as protecting renal function, promoting microcirculation, scavenging oxygen free radicals, and improving vascular endothelial function (Shen et al., 2013; Yu et al., 2015). Furthermore, it has been reported that salvianolate could treat atherosclerosis *via* regulating the inflammation at cytokine and cell levels (Meng et al., 2014); it could improve cerebral ischemic injury and the mechanism may be related to the activation of the vascular endothelial growth factor (VEGF) and BDNF-TrkB-CREB signaling pathway (He et al., 2015); it protected hepatocytes from oxidative stress by attenuating mitochondrial injury (Zhao et al., 2016). Hence, salvianolate has been shown to have significant efficacy in various diseases.

Recently, numerous clinical studies have identified the benefits of salvianolate in the treatment of DN. In animal models, Qi et al. (2012) showed that its application improved the levels of urine protein and serum creatinine, and changed the activity of lactate dehydrogenase (LDH) and superoxide dismutase (SOD), thereby indicating protective effects on renal function. However, there is still short of systematic evidence to confirm its clinical effect. Thus, we conducted a meta-analysis to evaluate the efficacy and safety of salvianolate as a complementary therapy to treat DN.

## Materials and Methods

### Literature Selection

The following electronic databases were searched: Pubmed, Web of Science, the Cochrane Library, China National Knowledge Infrastructure(CNKI), Wanfang Data knowledge service platform (Wanfang Data), Chinese Scientific Journal Database (VIP), and China Biology Medicine Disc (SinoMed). The retrieval time was from the creation of each database until November 30, 2019. To avoid omission, the retrieval scheme contained subject words with free words, keywords, or full text. We searched Chinese words such as “Danshenduofensuanyan”, “Danshenduofensuanyan Injection”, “Tangniaobing Shenbing”, “Tangniaobing Shensunhai”, and English words such as “Salvianolate”, “Salvia Polyphenola”, “Salvia Miltiorrhiza Polyphenolate”, “Diabetic Nephropathy”, “Diabetic Kidney Disease”, “Diabetic Renal Damage”, “DN”, and “DKD” (**Supplementary Tables 1** and **2**). The retrieval language was restricted to Chinese and English. We also used manual searches to ensure the integrity of the included studies.

### Inclusion Criteria

Studies should meet the criteria as follows: (1) Patients were diagnosed with DN based on the World Health Organization (WHO) diagnostic standard and Mogensen staging criteria (Miyauchi et al., 2009; Ge and Xu, 2013), with the exclusion of those with primary kidney diseases, systemic infectious diseases, malignant tumors, and other serious organ dysfunction diseases; (2) The treatment group was administered salvianolate injection combined with conventional treatment; (3) The control group was given conventional treatment including lifestyle changes, interventions of diet habits, administration of hypoglycemic drugs, control of blood pressure, and regulation of dyslipidemia; (4) One or some related outcomes, including renal function (serum creatinine, blood urea nitrogen, urinary albumin excretion rate, 24-hour urinary protein, and albumin-to-creatinine ratio), inflammatory factors (hypersensitive C-reactive protein and Interleukin-6), oxidative stress indicators (malondialdehyde and superoxide dismutase), clinical efficacy, and adverse reactions were reported in the studies; (5) Only published randomized controlled trials (RCTs) were included, irrespective of blinding; (6) Remedy in the two groups continued for 2 weeks or longer; (7) Salvianolate was provided by Shanghai Green Valley Pharmaceutical Co., Ltd (Shanghai, China).

### Exclusion Criteria

The following studies were excluded: (1) those that were unpublished; (2) those with non-randomized controlled trials or significant defect in experimental design; (3) annimal experiments, case reports, reviews, or experience summaries; (4) those with inconsistency in conventional therapies given in the two groups; and (5) those that failed to obtain the sufficient available data.

### Quality Evaluation

Methodological quality was evaluated using the bias risk assessment tool from Cochrane Handbook (version 5.0), which includes the following 6 aspects: (1) random allocation methods; (2) allocation concealment; (3) blinding; (4) integrity of outcome data; (5) selective reporting; and (6) other sources of bias. All the above were classified as “low risk”, “high risk”, or “unclear risk” (Yang, 2018).

### Data Extraction

Two evaluators independently screened the collected studies based on the inclusion and exclusion criteria previously mentioned. According to the pre-designed data extraction form, the relevant information collected were title, author’s name, publication year, research object, research method, treatment intervention, outcomes, and adverse reactions. Any disagreement during evaluation was solved by negotiation. If such disagreement was not resolved, a third researcher joined the discussion and made the final decision.

### Data Analysis

RevMan5.3 provided by the Cochrane collaboration network was used to perform statistical analysis for this meta-analysis (Ma, 2018). The selection of effect quantity includes the following: (1) Risk ratio (RR) was chosen as the dichotomous variable; (2) Mean difference (MD) and the standard mean difference (SMD) were expressed as the continuous variables; and (3) 95% confidence interval (CI) was used for interval estimation. Heterogeneity was determined using chi-squared test and the I^2^ statistic. If I^2^ was less than 50%, it represented homogeneity and a fixed effect model was selected. Otherwise, a random effect model was used, and the heterogeneity of the included studies should be analyzed. Subgroup analysis based on the research characteristics was carried out to explore the source of heterogeneity. Moreover, sensitivity analysis was used to assess the stability of the meta-analysis results. If the number of included studies were more than ten, potential publication bias will be detected using funnel plot. The *P-*value less than 0.05 was considered statistically significant.

## Results

### Search Results

**Figure 1:**
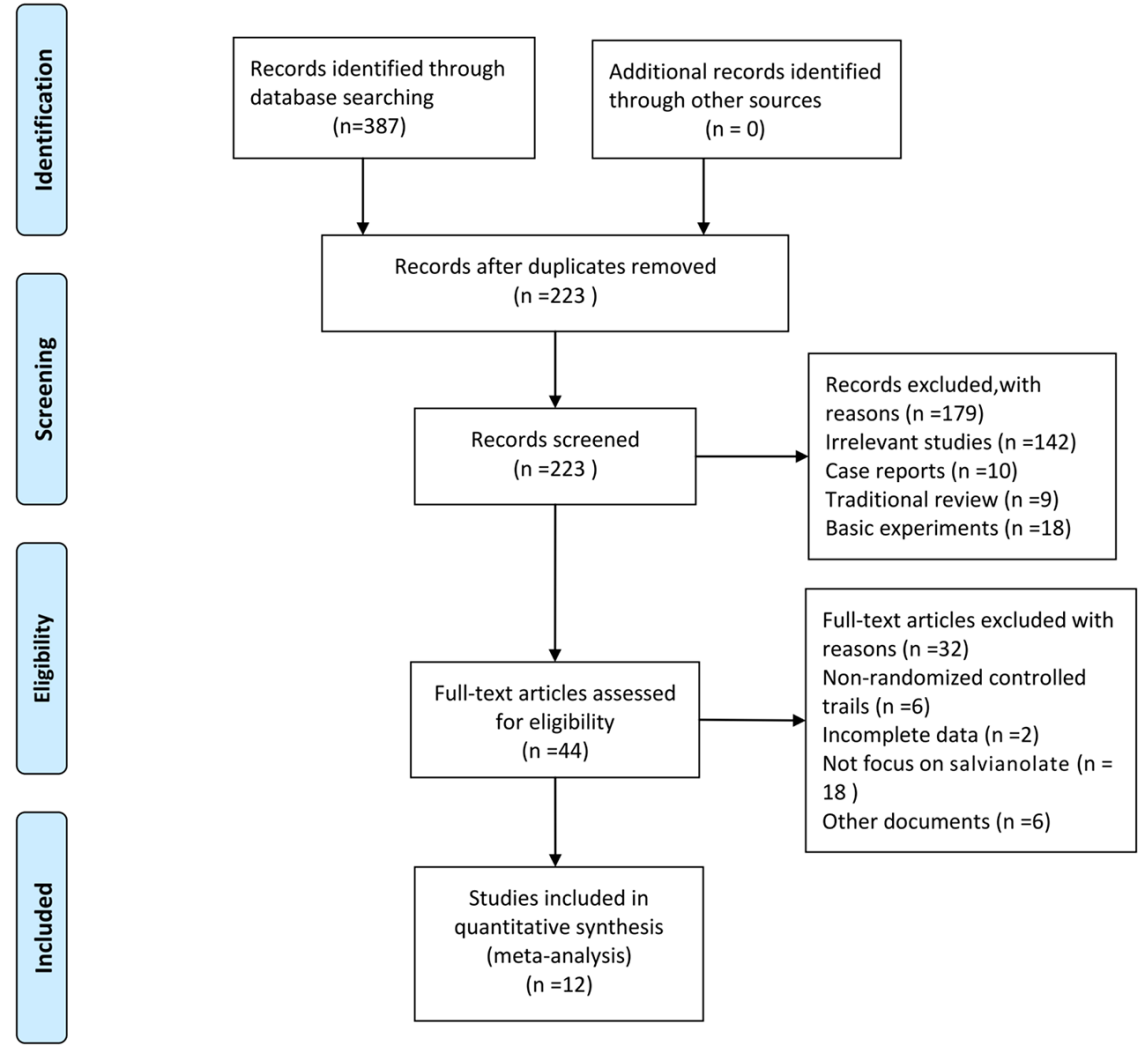
Flow diagram of literature screening.

Initially, 387 articles were obtained. We used the Endnote software for document management, and 164 duplicated articles were eliminated. From the title and abstract, 179 articles were excluded because they were irrelevant studies (n=142), case reports (n=10), traditional reviews (n=9), and basic experiments (n=18). After assessing the full texts carefully, 32 out of the remaining 44 articles were removed, which included non-randomized control trials (n=6), trials with incomplete data (n=2), studies not focusing on salvianolate (n=18), and other documents (n=6). Finally, 12 articles (Xiong et al., 2013; Liu et al., 2014; Lu et al., 2014; Xu, 2014; Zhang and Li, 2014; Ma and Zhao, 2015; Zhang et al., 2015; Zhao et al., 2015; Chen et al., 2018; Fu and Shao, 2018; Chen et al., 2019; Liang and Wang, 2019) were included for the meta-analysis. The screening process is presented in **Figure 1**.

### Study Characteristics and Quality Evaluation

**Figure 2:**
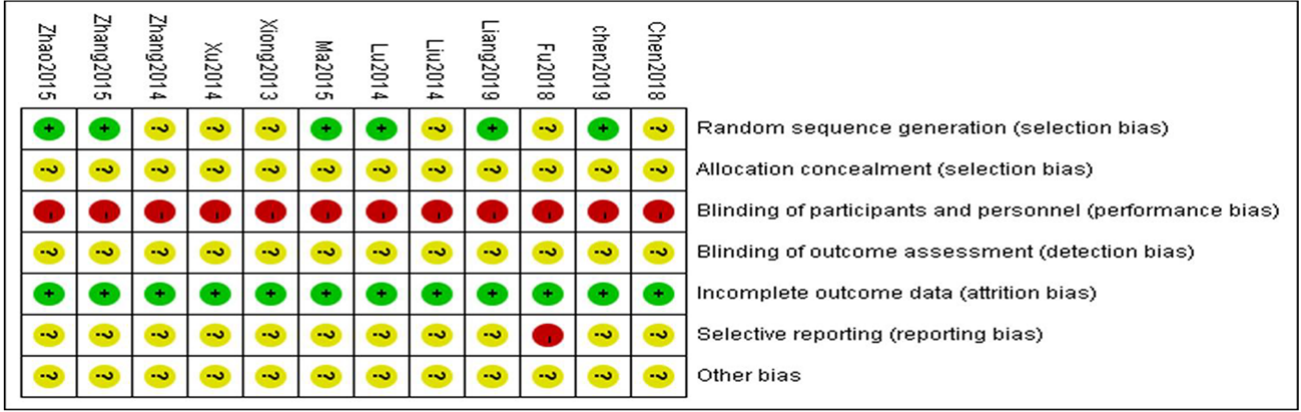
Graphs for risk of bias.

From the 12 randomized controlled trials (Xiong et al., 2013; Liu et al., 2014; Lu et al., 2014; Xu, 2014; Zhang and Li, 2014; Ma and Zhao, 2015; Zhang et al., 2015; Zhao et al., 2015; Chen et al., 2018; Fu and Shao, 2018; Chen et al., 2019; Liang and Wang, 2019) selected, there were 1,030 patients with 514 in the control group and 516 in the treatment group. The basic characteristics of the 12 RCTs are summarized in **Table 1**. The methodological quality of the studies included was low. Although all the studies used random grouping, only 6 (Lu et al., 2014; Ma and Zhao, 2015; Zhang et al., 2015; Zhao et al., 2015; Chen et al., 2019; Liang and Wang, 2019) described the specific grouping methods. None of the studies reported blinding or allocation concealment. The quality assessment is shown in **Figure 2**.

**Table 1:** Basic characteristics of inclusion in the study.

Study	Sample size	Sex	Age(Years)	Duration of	Course of	Intervention			Outcomes	Adverse
	T/C	(M/F)	Range,mean	diabetes(Years)	treatment	T		C		reactions
Xiong et al., 2013	30/30	T: 17/13 C: 18/12	T: 55.8 ± 7.4 C: 56.5 ± 7.3	T: 7.7 ± 1.7 C: 7.6 ± 1.8	3 weeks	Salvianolate (200 mg, ivgtt, qd)+ conventional treament		Conventional treament	Scr, UAER	None
Zhang and Li, 2014	45/45	50/40	T: 48.9 ± 10.1 C: 49.4 ± 9.6	T: 8.49 ± 1.6 C: 8.51 ± 1.4	2 weeks	Salvianolate (100 mg, ivgtt, qd)+ conventional treament		Conventional treament	SOD, MDA, ACR	Not reported
Xu, 2014	30/30	T: 18/12 C: 19/11	T: 56.34 ± 6.06 C: 55.81 ± 7.24	T: 6.25 ± 5.07 C: 6.69 ± 4.62	4 weeks	Salvianolate (200 mg, ivgtt, qd)+ conventional treament		Conventional treament	Scr, UAER	Not reported
Liu et al., 2014	30/30	T: 14/16 C: 17/13	T: 65.12 ± 9.83 C: 65.4 ± 9.6	T: 12.91 ± 2.12 C: 12.42 ± 2.28	2 weeks	Salvianolate (200 mg, ivgtt, qd)+ conventional treament		Conventional treament	Scr, BUN, UAER	T: 1 case of Palpitations during infusion C: None
Lu et al., 2014	33/31	T: 16/17 C: 16/15	T: 56.16 ± 9.09 C: 54.70 ± 9.26	– –	2 weeks	Salvianolate (200 mg, ivgtt, qd)+ Insulin therapy		Insulin therapy	hs-CRP, IL-6	Not reported
Zhao et al., 2015	45/45	T: 24/21 C: 24/21	T: 63.4 ± 9.80 C: 63.0 ± 11.1	T: 7.1 ± 2.4 C: 7.3 ± 1.4	4 weeks	Salvianolate (200 mg, ivgtt, qd)+ conventional treament		Conventional treament	Scr, BUN, UAER, Clinical efficacy	None
Zhang et al., 2015	42/42	T: 23/19 C: 25/17	T: 39~72 C: 41~71	T: 5.8 ± 1.4 C: 5.7 ± 1.1	2 weeks	Salvianolate (200 mg, ivgtt, qd)+ conventional treament		Conventional treament	UAER, hs-CRP, IL-6	Not reported
Ma and Zhao, 2015	59/59	T: 29/30 C: 27/32	T: 53~78 C: 51~79	T: 12.3 ± 3.8 C: 12.5 ± 2.8	2 weeks	Salvianolate (200 mg, ivgtt, qd)+ conventional treament		Conventional treament	hs-CRP, IL-6	Not reported
Fu and Shao, 2018	38/38	T: 22/16 C: 18/20	T: 45~75 C: 42~74	T: 5.98 ± 2.27 C: 6.04 ± 2.03	2 weeks	Salvianolate (100 mg, ivgtt, qd)+ Irbesartan (150 mg, po, qd)		Irbesartan (150mg, po, qd)	Scr, BUN, Clinical efficacy, hs-CRP, IL-6, MDA, SOD, 24h Upro, ACR	None
Chen et al., 2018	60/60	T: 39/21 C: 38/22	T: 68.82 ± 8.63 C: 68.45 ± 8.35	T: 6.54 ± 2.64 C: 6.65 ± 2.15	2 weeks	Salvianolate (200 mg, ivgtt, qd)+ Losartan potassium (50 mg, po, qd)		Losartan potassium (50mg, po, qd)	Scr, BUN, Clinical efficacy, hs-CRP, IL-6, 24h Upro	Not reported
Liang and Wang, 2019	47/47	T: 27/20 C: 26/21	T: 57.34 ± 6.28 C: 57.15 ± 6.34	T: 4.87 ± 2.41 C: 4.95 ± 2.36	2 weeks	Salvianolate (200 mg, ivgtt, qd)+ Irbesartan (150 mg, po, qd)		Irbesartan (150mg, po, qd)	Scr, BUN, Clinical efficacy, SOD, MDA, 24h Upro	Not reported
Chen et al., 2019	57/57	T: 33/24 C: 34/23	T: 58.41 ± 3.87 C: 57.25 ± 3.64	T: 4.93 ± 1.21 C: 4.85 ± 1.12	2 weeks	Salvianolate (200 mg, ivgtt, qd)+ conventional treament	conventional treament		Scr, BUN, Clinical efficacy, UAER	None

T, treatment group; C, control group; M, male; F, female; Scr, serum creatinine; UAER, urinary albumin excretion rate; BUN, blood urea nitrogen; SOD, superoxide dismutase; MDA, malondialdehyde; hs-CRP, hypersensitive C-reactive protein; IL-6, Interleukin-6; 24h Upro, 24-hour urinary protein; ACR, albumin-to-creatinine ratio.

### Outcomes

#### Effect on Renal Function

##### Serum Creatinine (Scr)

**Figure 3:**
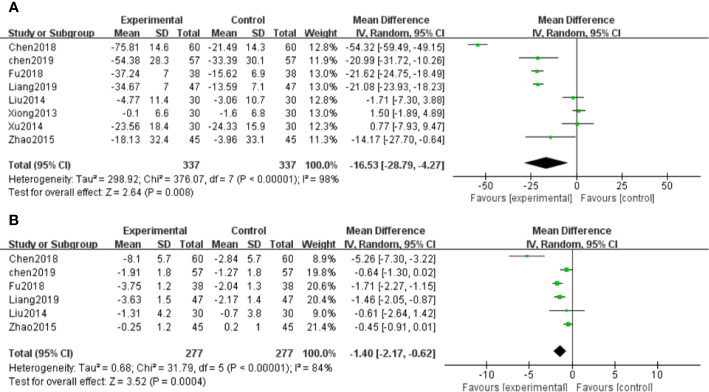
Forest plot for serum creatinine (Scr) between the treatment group and control group **(A)**. Forest plot for blood urea nitrogen (BUN) between the treatment group and control group **(B)**.

Eight RCTs measured Scr levels (Xiong et al., 2013; Liu et al., 2014; Xu, 2014; Zhao et al., 2015; Chen et al., 2018; Fu and Shao, 2018; Chen et al., 2019; Liang and Wang, 2019) to assess the renal function in DN patients. After heterogeneity test (*P* < 0.1, I^2^ = 98%), a random effect model was used to pool the data. The meta-analysis indicated that the intervention of salvianolate combined with western medicine performed better than using western medicine alone in terms of decreased Scr levels [MD=–16.53, 95% CI (–28.79, –4.27), Z=2.64, *P*=0.008] (**Figure 3A**).

##### Blood Urea Nitrogen (BUN)

Six studies (Liu et al., 2014; Zhao et al., 2015; Chen et al., 2018; Fu and Shao, 2018; Chen et al., 2019; Liang and Wang, 2019) assessed the BUN levels of DN patients. Due to significant heterogeneity (*P* < 0.1, I^2^ = 84%), a random effect model was chosen to pool the data. The results showed that patients with salvianolate plus western drugs demonstrated significantly decreased BUN levels than those with western drugs alone [MD=–1.40, 95% CI (–2.17, –0.62), Z=3.52, *P*=0.0004] (**Figure 3B**).

##### Urinary Albumin Excretion Rate (UAER)

**Figure 4:**
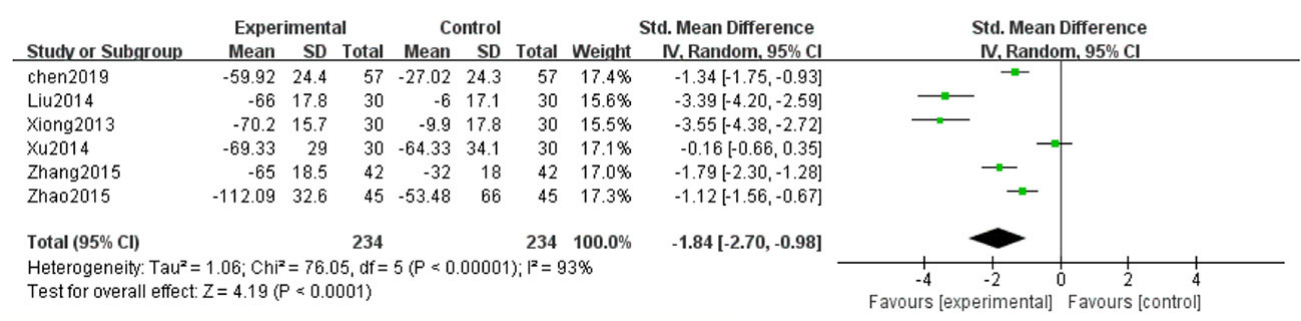
Forest plot for urinary albumin excretionrate (UAER) between the treatment group and control group.

Six studies (Xiong et al., 2013; Liu et al., 2014; Xu, 2014; Zhang et al., 2015; Zhao et al., 2015; Chen et al., 2019) reported UAER in the treatment of DN. Because of high heterogeneity (*P* < 0.1, I^2^ = 93%), a random effect model was adopted to pool the data. The meta-analysis revealed that compared with the control group, the application of salvianolate combined with western medicine had more significantly reduced UAER [SMD=–1.84, 95% CI (–2.70, –0.98), Z=4.19, *P* < 0.0001] (**Figure 4**).

##### 24-h Urinary Protein (24h Upro)

**Figure 5:**
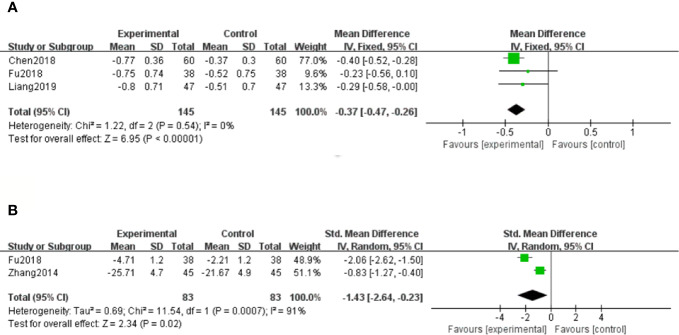
Forest plot for 24-hour urinary protein (24h Upro) between the treatment group and control group **(A)**. Forest plot for albumin-to-creatinine ratio (ACR) between the treatment group and control group **(B)**.

Three studies (Chen et al., 2018; Fu and Shao, 2018; Liang and Wang, 2019) showed 24h Upro results of DN patients. With the result of homogeneity after test (*P*=0.54, I^2^ = 0%), we chose a fixed effect model. The meta-analysis suggested that salvianolate combined with western medicine was more effective than the control group in decreasing 24h Upro [MD=–0.37, 95% CI (–0.47, –0.26), Z=6.95, *P* < 0.00001] (**Figure 5A**).

##### Albumin-to-Creatinine Ratio (ACR)

Only two studies (Zhang and Li, 2014; Fu and Shao, 2018) out of twelve reported the ratio of ACR at the end of the treatment. Because of high heterogeneity (*P* < 0.1, I^2^ = 91%), a random effect model was selected to pool the data. The meta-analysis revealed that compared with the control group, patients with salvianolate plus western drugs showed more advantages in reducing the ratio of ACR [SMD=–1.43, 95% CI (–2.64, –0.23), Z=2.34, *P*=0.02] (**Figure 5B**).

#### Effect on Inflammatory Factors

##### Hypersensitive C-Reactive Protein (hs-CRP)

**Figure 6:**
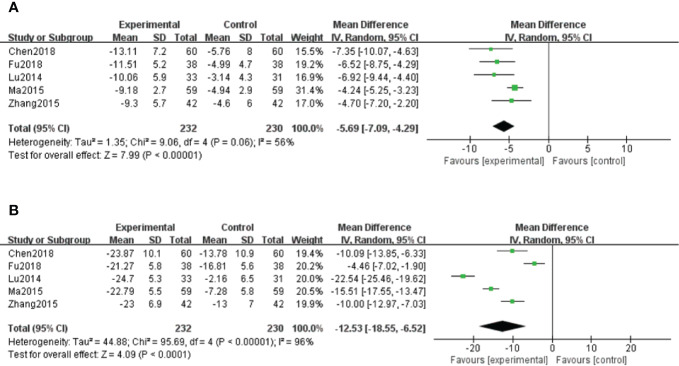
Forest plot for hypersensitive C-reactive protein (hs-CRP) between the treatment group and control group **(A)**. Forest plot for interleukin-6 (IL-6) between the treatment group and control group **(B)**.

A total of five studies (Lu et al., 2014; Ma and Zhao, 2015; Zhang et al., 2015; Chen et al., 2018; Fu and Shao, 2018) measured hs-CRP levels. Because of moderate heterogeneity (*P* < 0.1, I^2^ = 56%), a random effect model was adopted for data analysis. The meta-analysis indicated that hs-CRP levels in patients treated with salvianolate plus western drugs were significantly lower than those in the control group [MD=–5.69, 95% CI (–7.09, –4.29), Z=7.99, *P* < 0.00001] (**Figure 6A**).

##### Interleukin-6 (IL-6)

A total of five studies (Lu et al., 2014; Ma and Zhao, 2015; Zhang et al., 2015; Chen et al., 2018; Fu and Shao, 2018) reported IL-6 results. Because of high heterogeneity (*P* < 0.1, I^2^ = 96%), a random effect model was used for data analysis. The meta-analysis showed significantly decreased levels of IL-6 in salvianolate combined with western medicine compared with the single-use western medicine [MD=–12.53, 95% CI (–18.55, –6.52), Z=4.09, *P* < 0.0001] (**Figure 6B**).

#### Effect on Oxidative Stress Indicators

##### Superoxide Dismutase (SOD)

**Figure 7:**
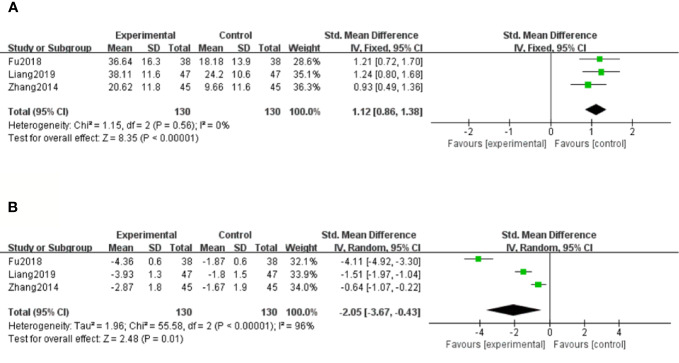
Forest plot for superoxide dismutase (SOD) between the treatment group and control group **(A)**. Forest plot for malondialdehyde (MDA) between the treatment group and control group **(B)**.

There were three studies (Zhang and Li, 2014; Fu and Shao, 2018; Liang and Wang, 2019) that assessed SOD levels. With the result of homogeneity after test (*P*=0.56, I^2^ = 0%), we chose a fixed effect model. The meta-analysis demonstrated that compared with the control group, significantly increased SOD levels were observed in salvianolate plus western drugs [SMD=1.12, 95% CI (0.86, 1.38), Z=8.35, *P* < 0.00001] (**Figure 7A**).

##### Malondialdehyde (MDA)

There were three studies (Zhang and Li, 2014; Fu and Shao, 2018; Liang and Wang, 2019) that measured MDA levels. Because of considerable heterogeneity (*P* < 0.1, *I^2^ =* 96%), a random effect model was adopted for data analysis. The meta-analysis revealed that the combination treatment of salvianolate and western medicine had more significantly reduced MDA levels compared with western medicine monotherapy [SMD=–2.05, 95% CI (–3.67, –0.43), Z=2.48, *P*=0.01] (**Figure 7B**).

### Clinical Efficacy

**Figure 8:**
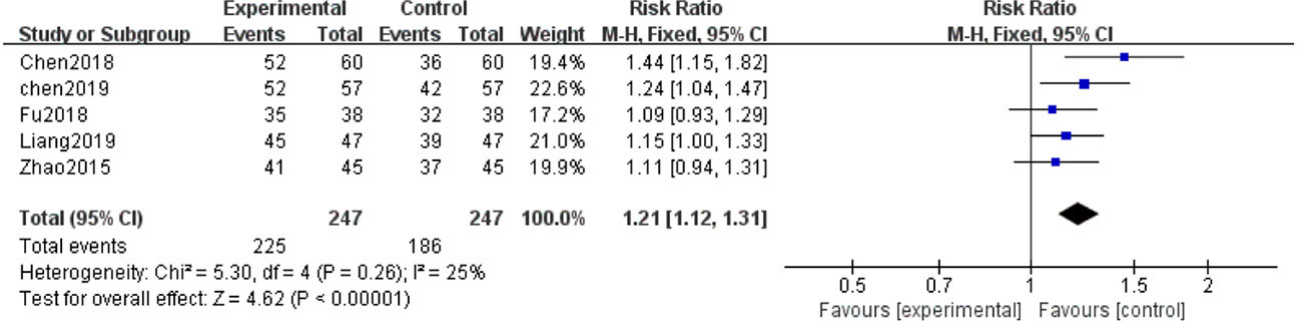
Forest plot for clinical efficacy between the treatment group and control group.

Five of the 12 studies (Zhao et al., 2015; Chen et al., 2018; Fu and Shao, 2018; Chen et al., 2019; Liang and Wang, 2019) evaluated clinical efficacy. With the result of homogeneity after test (*P*=0.26, I^2^ = 25%), we used a fixed effect model. The meta-analysis demonstrated that the combination of salvianolate and western medicine was superior to the single-use western medicine in improving clinical efficacy [RR=1.21, 95% CI (1.12,1.31), Z=4.62, *P* < 0.00001] (**Figure 8**).

### Adverse Reactions

Five out of the 12 trials (Xiong et al., 2013; Liu et al., 2014; Zhao et al., 2015; Fu and Shao, 2018; Chen et al., 2019) described adverse reactions. Only one (Liu et al., 2014) reported a case with palpitation during salvianolate injection, and the symptoms of which disappeared after adjusting the infusion rate. The other four trials (Xiong et al., 2013; Zhao et al., 2015; Fu and Shao, 2018; Chen et al., 2019) found no adverse reactions in the treatment group or control group. However, because the remaining trials did not provide any details on adverse effects, the safety of salvianolate still needs to be treated with caution in the future.

### Subgroup Analysis

According to the stage of Mogensen, we excluded the study (Chen et al., 2018) since they did not clearly provide the staging results of DN patients. And the remaining 11 RCTs were divided into two subgroups: stage III group (Xiong et al., 2013; Liu et al., 2014; Lu et al., 2014; Xu, 2014; Zhang and Li, 2014; Ma and Zhao, 2015; Zhang et al., 2015; Zhao et al., 2015), and stage III-IV group (Fu and Shao, 2018; Liang and Wang, 2019; Chen et al., 2019). To explore the source of heterogeneity, subsequent subgroup analyses were conducted for Scr, BUN and UAER, as they were crucial indicators on the evauation of renal function. The pooled data are shown in **Table 2**. The results indicated significant differences between the group treated with salvianolate plus western drugs and that with single-use western drugs for each subgroup in terms of the stage of DN (**Supplementary Figures 1–3**). However, for Scr, there was no significant difference in stage III [MD=–0.97, 95% CI (–5.32, 3.38), *P*=0.66]. Meanwhile, the declined heterogeneity on Scr and BUN demonstrated that the stage of DN may be a cause of heterogeneity. Nevertheless, for UAER, the heterogeneity was not removed from subgroup analysis.

**Table 2:** Subgroup analyses for Scr, BUN, and UAER.

Outcome	Stage of DN	n	MD/SMD(95% CI)	I^2^(%)	Z	*P*
Scr	stage III	4	–0.97 (–5.32,3.38)	44	0.44	0.66
	stage III-IV	3	–21.31 (–23.38, –19.25)	0	20.21	<0.00001
BUN	stage III	2	–0.46 (–0.90, –0.01)	0	2.01	0.04
	stage III-IV	3	–1.29 (–1.90, –0.68)	67	4.16	<0.0001
UAER	stage III	5	–1.96 (–3.09, –0.83)	95	3.40	0.0007
	stage III-IV	1	–1.34 (–1.75, –0.93)	Not applicable	6.45	<0.00001

Scr, serum creatinine; BUN, blood urea nitrogen; UAER, urinary albumin excretion rate.

### Sensitivity Analysis

Sensitivity analyses were carried out for outcomes with heterogeneity by removing the included studies one by one, to observe whether the results were significantly changed (**Table 3**). We found that heterogeneity still existed and the outcomes after the removal of each study were similar, indicating that the results were not affected by any individual study and had relatively good stability, except for hs-CRP results of Ma and Zhao (2015) and MDA results of Liang and Wang (2019). For hs-CRP results, heterogeneity dropped from 56% to 0% when the study Ma and Zhao (2015) was excluded. Therefore, this RCT may be a source of considerable heterogeneity. One of the possible explanations would be the longest course of disease that influenced the sensitivity to drugs during treatment. For MDA results, the *P*-value greater than 0.05 became insignificant when the study Liang and Wang (2019) was eliminated. Despite the overall significant *P*-value, the results of MDA were not robust enough and need to be further verified.

**Table 3:** Sensitivity analyses for Scr, BUN, UAER, ACR, hs-CRP, IL-6, and MDA.

Outcome	Study		Data with study removed					
			MD/SMD (95% CI)	I^2^		Z		*P*
Scr								
	Xiong et al., 2013		–22.17 (–23.94, –20.41)	97		24.67		<0.00001
	Xu, 2014		–17.74 (–19.33, –16.15)	98		21.88		<0.00001
	Liu et al., 2014		–18.45 (–20.08, –16.82)	98		22.21		<0.00001
	Zhao et al., 2015		–17.18 (–18.76, –15.61)	98		21.40		<0.00001
	Fu and Shao, 2018		–15.65 (–17.45, –13.84)	98		16.99		<0.00001
	Chen et al., 2018		–13.40 (–15.04, –11.76)	96		16.02		<0.00001
	Liang and Wang, 2019		–15.45 (–17.32, –13.58)	98		16.19		<0.00001
	Chen et al., 2019		–17.06 (–18.64, –15.48)	98		21.16		<0.00001
BUN								
	Liu et al., 2014		–1.49 (–2.32, –0.65)	87		3.48		0.0005
	Zhao et al., 2015		–1.66 (–2.54, –0.77)	81		3.66		0.0003
	Fu and Shao, 2018		–1.34 (–2.27, –0.42)	84		2.84		0.005
	Chen et al., 2018		–1.03 (–1.62, –0.44)	74		3.45		0.0006
	Liang and Wang, 2019		–1.44 (–2.42, –0.45)	87		2.85		0.004
	Chen et al., 2019		–1.62 (–2.58, –0.66)	87		3.32		0.0009
UAER								
	Xiong et al., 2013		–1.52 (–2.32, –0.71)	92		3.71		0.0002
	Xu, 2014		–2.17 (–3.01, –1.34)	91		5.10		<0.00001
	Liu et al., 2014		–1.54 (–2.36, –0.72)	92		3.68		0.0002
	Zhao et al., 2015		–2.00 (–3.09, –0.92)	95		3.61		0.0003
	Zhang et al., 2015		–1.86 (–2.92, –0.80)	95		3.45		0.0006
	Chen et al., 2019		–1.96 (–3.09, –0.83)	95		3.40		0.0007
ACR								
	Zhang and Li, 2014		–2.06 (–2.62, –1.50)	Not applicable		7.20		<0.00001
	Fu and Shao, 2018		–0.83 (–1.27, –0.40)	Not applicable		3.79		0.0002
hs-CRP							
	Lu et al., 2014		–5.43 (–6.94, –3.92)	56		7.05		<0.00001
	Zhang et al., 2015		–5.98 (–7.73, –4.24)	67		6.72		<0.00001
	Ma and Zhao, 2015		–6.34 (–7.58, –5.11)	0		10.05		<0.00001
	Fu and Shao, 2018		–5.52 (–7.14, –3.90)	59		6.68		<0.00001
	Chen et al., 2018		–5.34 (–6.75, –3.94)	51		7.46		<0.00001
IL-6								
	Lu et al., 2014		–10.05 (–15.30, –4.79)	93		3.75		0.0002
	Zhang et al., 2015		–13.16 (–20.66, –5.67)	97		3.44		0.0006
	Ma and Zhao, 2015		–11.77 (–19.79, –3.75)	96		2.88		0.004
	Fu and Shao, 2018		–14.60 (–19.98, –9.22)	93		5.32		<0.00001
	Chen et al., 2018		–13.12 (–20.33, –5.92)	96		3.57		0.0004
MDA								
	Zhang and Li, 2014		–2.78 (–5.33, –0.23)	97		2.14		0.03
	Fu and Shao, 2018		–1.07 (–1.91, –0,22)	86		2.48		0.01
	Liang and Wang, 2019		–2.36 (–5.75,1.04)	98		1.36		0.17

Scr, serum creatinine; BUN, blood urea nitrogen; UAER, urinary albumin excretion rate; ACR, albumin-to-creatinine ratio; hs-CRP, hypersensitive C-reactive protein; IL-6, Interleukin-6; MDA, malondialdehyde.

### Publication Bias

Due to the lack of studies (< 10), we did not perform the publication bias analysis.

## Discussion

Salvianolate, derived from the extract of *Salvia miltiorrhiza*, is the first Chinese medicine preparation with pharmacokinetic parameters in humans and has the highest content of active ingredients in *Salvia miltiorrhiza* preparations (Jiang et al., 2013). Modern clinical studies demonstrated that salvianolate is mainly used in cardiocerebral vascular diseases, because its main component salvia magnesium acetate has anti-lipid peroxidation properties and can improve endothelial function. Furthermore, it plays an important role in the prevention and treatment of some metabolic diseases (Fan, 2012; Zhang et al., 2013; Niu and Niu, 2016). Recently, studies on the active components of TCM received increasing interest, particularly those that meet the new therapeutic targets of DN.

DN is a chronic disease characterized by the thickening of the glomerular basement membrane and the accumulation of extracellular matrix on the basis of renal hyperfiltration and highperfusion, eventually leading to renal failure (Mauer et al., 1984). According to the theory of TCM, microvascular diseases belong to the category of “collateral disease”. Following the viewpoints of “chronic diseases transforming to collaterals” (“jiu bing ru luo”) and “ chronic diseases being stasis” (“jiu bing cheng yu”), it is particularly important to promote blood circulation and eliminate blood stasis (Hu et al., 2016). Coincidentally, salvianolate activates blood circulation, removes stasis, and dredges pulse. These effects not only conform with the TCM treatment principle, but have also been supported by modern pharmacology (Wu et al., 2010). Salvianolate has antioxidative properties, inhibits inflammation, and improves microcirculation, thereby exhibiting protective effects to kidneys. These pharmacological activities exert their effects through multiple targets and links (Hu et al., 2011).

To our knowlwdge, this is the first systematic review to comprehensively assess the relation between salvianolate and DN. Our meta-analysis included 12 RCTs with a total of 1030 patients suffering from DN. According to the pooled data analysis, compared with western medicine monotherapy, the combination of salvianolate and western medicine is more effective in reducing Scr, BUN, UAER, 24h Upro, ACR, hs-CRP, IL-6, and MDA levels, and improving SOD levels and clinical efficacy. Thus, all these results indicated that the combination therapy had a stronger effect in treating DN, as its action on protecting renal function, inhibiting inflammation, and performing antioxidation.

Complicated pathogenesis makes diagnosis and treatment of DN difficult. Fortunately, existing biomarkers can help understand its progression and drug therapeutic effects. Among the multiple biomarkers for evaluating DN, improved renal function reflects the drug’s kidney-protective mechanism to a certain extent. Scr and BUN are typical indicators of renal function and provide important information for kidney injury, of which Scr is also recommended by Kidney Disease Improving Global Outcomes (KDIGO) guideline (Xu et al., 2016; Zhu et al., 2020). In addition to reflecting the function of glomerular filtration, Scr and BUN levels can also indicate improvement in renal prognosis (Low et al., 2018). The reduction in their levels from our meta-analysis illustrated that salvianolate may paly a part in renoprotection. However, high heterogeneity of the results cannot be ignored. Subgroup analysis of Scr according to DN stage found no difference in stage III. This might be explained by an insignificant change with Scr levels in early DN. Besides, the stage of DN may be one of the sources because of the declined heterogeneity for both Scr and BUN in subgroup analyses. Proteinuria is another clinical hallmark that contributes to progression of DN (Wolf and Ziyadeh, 2007). It is thought to be more specific than total proteinuria for a glomerular source, and is often used as the standard for defining the presence of DN (Campbell et al., 2003). Earlier studies have showed that microalbuminuria (> 300 mg/24 h of albuminuria) is predictive of the further development of end-stage renal failure and cardiovascular diseases (Fineberg et al., 2013). Consequently, treatments that reduce proteinuria can preserve renal function in DN patients. In this study, UAER and 24h Upro as the major indexes of proteinuria were chosen for data analysis. Our results revealed that the combined application of salvianolate and western medicine had better efficacy in decreasing UAER and 24h Upro. However, we failed to find the cause of heterogeneity in UAER after conducting subgroup analysis and sensitivity analysis. Possible reasons may be related to factors such as age, sex, type of diabetes, disease course, and treatment duration. Although no heterogeneity existed in 24h Upro, the accuracy for collecting 24-hour urine was poor, which may increase the risk of bias. Thus, in clinical practice, the detection of ACR can be performed with random urine which reduces errors, was recommended to estimate renal function (Zhao, 2020). The results of our study showed significant reduced ACR in salvianolate and western medicine combination. Despite the considerate heterogeneity, it is hard to investigate the sources owing to the small number of patients. Thus, trials with larger sample sizes are required.

Furthermore, experimental research showed that patients affected with DN have varying degrees of inflammatory response and oxidative stress, whose mechanisms can serve as potential targets for long-term DN therapies (Brenneman et al., 2016). Potential biomarkers were emerging during the last years. hs-CRP is an acute phase protein sensitive to inflammation. In DN patients, the increase in hs-CRP levels is associated with increased IL-6 synthesis, because persistent hyperglycemia can stimulate islet cells to produce IL-6, which then acts on the liver and secretes hs-CRP in large quantities (Wang et al., 2019). SOD is the first line of defense against reactive oxygen species, which promote superoxide anion radicals to damage kidney tissues (Lu and Yin, 2019). MDA is a metabolite of prostaglandins that is generated through lipid peroxidation and increased free radicals caused by hyperglycemia and hyperlipidemia (Susztak et al., 2006). Based on the 12 studies, we found that the salvianolate combined with western medicine exerts beneficial effects in controlling inflammation and antioxidation. Due to heterogeneity, sensitive analyses were conducted in terms of hs-CRP, IL-6, and MDA. Unfortunately, the origin of heterogeneity remained unknown for IL-6. However, for hs-CRP, we found the cause may be linked to the study Ma and Zhao (2015) with the longest diabetes duration. For MDA, the results of insignificant *P*-value with the removed study Liang and Wang (2019) was not sufficient to draw a conclusion because of the small sample. Therefore, the effects of salvianolate in combination with western medicine on DN should be interpreted with caution. Moreover, salvianolate seems to be safe because of the absence of evident adverse reactions during treatment. Nevertheless, only 41.7% of the studies reported adverse reactions, the safety of salvianolate still needs to be validated in the future clinical trials.

### Limitations

Limitations in our meta-analysis should be considered as follows: First of all, literatures were included with low quality and small sample sizes. And the research methods were not reported in details, thereby making bias risk assessment difficult. Particularly, none of the studies provided any detail on single or double blinding, and allocation concealment, which indicated poor quality of methodology and led to high risk of selection and measurement bias. Secondly, although we adopted an adequate search strategy to minimize publication bias, some potential biases may still exist because of language restriction. Thirdly, drug safety is a key factor in clinical applications, but only five RCTs described adverse reactions or events. Therefore, the safety of using salvianolate should be validated in future to bring more convincing evidence. Fourthly, all the involved DN patients were in stage III ang IV, the effect of salvianolate on end-stage DN is uncertain and needs to be proved. Besides, none of the studies reported end-point outcomes such as the incidence of ESRD, fatality rate, and life quality, thus making the assessment of the long-term efficacy of salvianolate difficult, which will affect the further development of drugs. Lastly, the considerable heterogeneity observed mostly emerged in continuous variables such as Scr and BUN. It was difficult to find the real sources of heterogeneity, and we speculated that the causes may be relevant to sample size, research object, and the assessment method of outcomes.

## Conclusions

Findings from this meta-analysis illustrate that salvianolate combined with western medicine may be effective and safe in the treatment of DN. Because of the poor methodological quality and small sample sizes, further validation is essential. Therefore, we recommend the conduct of multicenter, large-sample, and randomized controlled double-blind trials to provide a more accurate and reliable evidence for clinical research.

## Author Contributions

YS proposed the subject and designed the protocol for this systematic review. SW, YL, and LG conducted literature screening and data extraction. LX, XZ, and YM performed statistical analysis. YS and SW drafted the manuscript. JS and QZ coordinated and inspected all aspects of the research design. All authors contributed to the revision of the manuscript and approved the final manuscript for submitting.

## Funding

This systematic review was supported by the National Natural Science Foundation of China (grant 81873274, 81603332).

## Conflict of Interest

The authors declare that the research was conducted in the absence of any commercial or financial relationships that could be construed as a potential conflict of interest.
